# STAT5 is required for lipid breakdown and beta-adrenergic responsiveness of brown adipose tissue

**DOI:** 10.1016/j.molmet.2020.101026

**Published:** 2020-05-28

**Authors:** Doris Kaltenecker, Katrin Spirk, Frank Ruge, Florian Grebien, Marco Herling, Anne Rupprecht, Lukas Kenner, Elena E. Pohl, Kristina M. Mueller, Richard Moriggl

**Affiliations:** 1 Institute of Animal Breeding and Genetics, University of Veterinary Medicine, 1210, Vienna, Austria; 2 Ludwig Boltzmann Institute for Cancer Research, 1090, Vienna, Austria; 3 Institute for Diabetes and Cancer (IDC), Helmholtz Zentrum München, German Research Center for Environmental Health, Neuherberg, Germany; 4 Institute for Medical Biochemistry, University of Veterinary Medicine, 1210, Vienna, Austria; 5 Laboratory of Lymphocyte Signaling and Oncoproteome, Department I of Internal Medicine, CIO Aachen-Bonn-Cologne-Duesseldorf, Excellence Cluster for Cellular Stress Response and Aging-Associated Diseases (CECAD), Center for Molecular Medicine Cologne (CMMC), University of Cologne, Cologne, Germany; 6 Unit of Physiology and Biophysics, University of Veterinary Medicine, 1210, Vienna, Austria; 7 Unit of Pathology of Laboratory Animals, University of Veterinary Medicine, Vienna, 1210, Vienna, Austria; 8 Department of Pathology, Medical University Vienna, 1090, Vienna, Austria; 9 Christian Doppler Laboratory for Applied Metabolomics, Medical University Vienna, 1090, Vienna, Austria

**Keywords:** JAK-STAT, β-adrenergic signalling, Thermogenesis, Temperature maintenance, ATGL, adipose triglyceride lipase, BAT, brown adipose tissue, cAMP, cyclic adenosine monophosphate, EWAT, epididymal WAT, FA, fatty acid, FCCP, carbonyl cyanide 4-(trifluoromethoxy) phenylhydrazone, GH, growth hormone, H&E, haematoxylin and eosin, HSL, hormone sensitive lipase, JAK, Janus kinase, mtDNA, mitochondrial DNA, NEFA, non-esterified fatty acids, PKA, protein kinase A, qPCR, quantitative real-time PCR, ScWAT, subcutaneous WAT, STAT, signal transducer and activator of transcription, UCP1, uncoupling protein 1, WAT, white adipose tissue

## Abstract

**Objective:**

Increasing energy expenditure through activation of brown adipose tissue (BAT) thermogenesis is an attractive approach to counteract obesity. It is therefore essential to understand the molecular mechanisms that control BAT functions. Until now several members of the Janus kinase (JAK) - signal transducer and activator of transcription (STAT) pathway have been implicated as being relevant for BAT physiology. However, whether the STAT family member STAT5 is important for the thermogenic property of adipose tissues is unknown. Therefore, we have investigated the role of STAT5 in thermogenic fat in this paper.

**Methods:**

We performed metabolic and molecular analyses using mice that harbor an adipocyte-specific deletion of *Stat5a/b* alleles.

**Results:**

We found that STAT5 is necessary for acute cold-induced temperature maintenance and the induction of lipid mobilization in BAT following β_3_-adrenergic stimulation. Moreover, mitochondrial respiration of primary differentiated brown adipocytes lacking STAT5 was diminished. Increased sensitivity to cold stress upon STAT5 deficiency was associated with reduced expression of thermogenic markers including uncoupling protein 1 (UCP1), while decreased stimulated lipolysis was linked to decreased protein kinase A (PKA) activity. Additionally, brown remodeling of white adipose tissue was diminished following chronic β_3_-adrenergic stimulation, which was accompanied by a decrease in mitochondrial performance.

**Conclusion:**

We conclude that STAT5 is essential for the functionality and the β-adrenergic responsiveness of thermogenic adipose tissue.

## Highlights

• Impaired temperature maintenance in mice deficient in adipocyte STAT5 after acute cold exposure.
• Blocked β_3_-adrenergic induction of lipolysis and PKA activity in BAT upon STAT5 deficiency.
• Reduced respiratory capacity in primary differentiated brown adipocytes lacking STAT5.
• Diminished brown remodeling of STAT5 deficient ScWAT after chronic β_3_-adrenergic stimulation.

## 1 Introduction

Brown adipose tissue (BAT) has a remarkable capacity for substrate oxidation [1]. Brown adipocytes are densely packed with mitochondria, in which they express uncoupling protein 1 (UCP1) that allows them to dissipate stored energy in the form of heat. UCP1 enables the cells to uncouple oxidative phosphorylation from ATP synthesis by catalysing a proton leak across the inner mitochondrial membrane [2]. This process accounts for the central function of BAT, which is heat production for temperature maintenance, also called non-shivering thermogenesis [3,4]. In addition to stored energy, glucose and non-esterified fatty acids (NEFA) are also imported from the circulation to provide substrates for continuous thermogenesis, suggesting that BAT is important in global nutrient partitioning and energy expenditure [5]. Since it has been shown that BAT exists in human adults [6,7], the strategy to increase BAT activity, and thereby energy expenditure, has become an attractive anti-obesity approach. The discovery of so-called ‘brite’ or beige cells extended the complexity of adipocyte biology [8]. In rodents, beige cells can be activated within white adipose tissue (WAT) depots by various stimuli, but most notably by prolonged cold exposure or β-adrenergic stimulation [9,10]. They have multilocular lipid droplets, abundant mitochondria, and express UCP1 [9].

The activation of thermogenic adipose tissue is mainly controlled by the sympathetic nervous system and β-adrenergic signalling [3]. Stimulation of β-adrenergic receptors leads to increased adenylyl cyclase activity and a consequent increase in the concentration of intracellular cyclic adenosine monophosphate (cAMP). Elevated cAMP levels activate protein kinase A (PKA), which stimulates lipolysis by directly phosphorylating hormone sensitive lipase (HSL) [11]. NEFAs that are released by this process, in addition to NEFAs that are imported from the circulation, can be used in mitochondrial β-oxidation to fuel thermogenesis and as substrates for the UCP1-mediated proton transport [1].

The field of BAT biology was advanced by the identification of signalling pathways that have an impact on transcriptional programs governing brown and beige fat cell function and development [12]. Among these, members of the Janus kinase (JAK)–signal transducer and activator of transcription (STAT) pathway were shown to control BAT physiology [[13], [14], [15]]. The transcription factors STAT5A and STAT5B (collectively referred to as STAT5) are considered as mainly activated by growth hormone (GH) and prolactin via JAK2 in adipose tissue and have been shown to be critical in adipocyte development [[16], [17], [18]]. Additionally, we demonstrated the importance of STAT5 for homeostasis of lipid metabolism in WAT, as disruption of adipocyte STAT5 signalling reduces basal lipolysis [19]. Although mice with adipocyte-specific STAT5 deficiency display increased adiposity, they are more sensitive to insulin and are further protected against insulin resistance associated with aging [19]. However, it is not clear whether STAT5 plays a role in thermogenic adipose tissue.

The goal of our study was to investigate the effects of adipocyte-specific STAT5 deficiency on the functionality of BAT and to explore whether STAT5 deficiency alters the browning capacity of WAT following chronic β-adrenergic stimulation by using an *in vivo* model.

## 2 Methods

### 2.1 Animal breeding, experimentation, and housing

Adipocyte-specific STAT5-deficient mice (*Stat5*^*Adipoq*^) were generated as previously described [19]. Mice were housed under standardised conditions (12h dark/12h light cycle). Unless stated otherwise, experiments were performed using 2-month-old male *Stat5*^*Adipoq*^ mice or Adipoq-Cre negative littermates (control, *Stat5* floxed) maintained on a C57BL/6N background and fed a standard diet *ad libitum*. Animal experimentation was approved by the institutional ethics and animal welfare committee and the national authority according to §26 of the Animal Experiments Act (Tierversuchsgesetz 2012). To evaluate temperature maintenance, mice were exposed to cold (4–5 °C) for the indicated time points. Chronic β-adrenergic stimulation was performed by i.p. CL316243 injections (1 mg/kg) once a day for 10 days. Control groups received vehicle (PBS) injections.

### 2.2 Histology

Formaldehyde-fixed tissues were dehydrated, paraffin-embedded, sliced, and stained with haematoxylin and eosin (H&E) using standard procedures and were analysed using light microscopy. For electron microscopy, mouse BAT was cut into 2-mm pieces and fixed in 1.6% glutaraldehyde overnight. Photographs were taken at a 4,000× magnification using a transmission electron microscope.

### 2.3 Quantitative mtDNA copy number determination

Mitochondrial copy number was determined as previously described [20]. DNA was isolated and purified from BAT using standard protocols. Mitochondrial DNA (mtDNA) was determined in relation to genomic DNA by quantitative real-time PCR (qPCR). Primers (see Supplementary Table 1) used for mitochondrial genes were against cytochrome c oxidase subunit 1 (*Co1*) and NADH dehydrogenase subunit 1 (*Nd1*), and primers for the nuclear genes were against NADH: ubiquinone oxidoreductase core subunit V1 (*Ndufv1*).

### 2.4 Metabolite measurements

β-ketones were measured directly from tail vein blood (glucometer: Abbott, Chicago, IL, USA). Plasma NEFA levels were determined using a NEFA-HR(2) kit (Wako Chemicals, Neuss, Germany).

### 2.5 RNA-Seq processing and bioinformatics data analysis

RNA was extracted using a RNeasy Lipid Tissue Mini Kit (Qiagen, Venlo, Netherlands). RNA-Seq 50 bp single-end libraries were sequenced on a HiSeq 3000 (Illumina, San Diego, CA, USA) machine, resulting in an average of 22 M reads per replicate. Alignments to genome version GRCm38_mm10 and the corresponding Ensgene annotation were accomplished with STAR (STAR_2.5.0b) using default parameters [21]. Expression level estimation was performed with featureCounts (version 1.5.0-p1) [22] with the ‘-t exon’ option. Differential expression analysis was performed using DESeq2 (1.16.1) [23]. The design consisted of five replicates for control and *Stat5*^*Adipoq*^ BAT from *ad libitum* fed male mice. Genes with low expression were filtered out if the size factor normalized row sum was ≤45. The functions ‘DESeq()’ and ‘results()’ were then applied with default parameters. Gene ontology analysis was performed with GO consortium [[24], [25], [26]] using significantly (adjusted P value < 0.05) up- and down-regulated genes as input. Full GO lists can be found at GEO. The data have been deposited to the GEO (GSE137678).

### 2.6 qPCR and Western blotting

RNA was extracted as described above, reverse transcribed, and the cDNA was subjected to qPCR using the CFX96 Real-Time System (Biorad, Hercules, CA, USA). Samples were run in duplicate. Primers are listed in Supplementary Table 1. Western blotting (15–30 μg protein loaded per lane) was performed using standard procedures. Blots were incubated with antibodies against ATGL (#2439), pSer563-HSL (#4139), HSL (#4107), UCP1 (#14670), Phospho-STAT1 (Y701) (58D6) (#9167); Phospho-STAT3 (Ser727) (#9134), pPKA Substrate (#9624; all from Cell Signaling), HSC70 (sc-7298 from Santa Cruz Biotechnology), Adiponectin (ab22554, Abcam), STAT3 (610189) and STAT5 (610191, both from BD). Antibodies were used at a 1:1000 dilution except for HSC70, which was used at 1:10,000.

### 2.7 *Ex vivo* measurement of lipolysis

Lipolysis of BAT explants was measured as described [27]. Explants were stimulated with 1 μM CL316243 (Tocris, Bristol, UK) or 500 ng/ml GH (Immunotools, Friesoythe, Germany).

### 2.8 Isolation, differentiation, and bioenergetics profiling of primary brown adipocytes

Stroma vascular cells were isolated from BAT of 4-5-week old mice and primary brown adipocytes were differentiated *in vitro* using standard protocols. A detailed description is available in the supplementary information. A mitochondrial stress test was performed using the Seahorse XFe96 extracellular flux analyser (Agilent, Santa Clara, CA, USA) as described [28]. Serial injections were performed with 5 μM oligomycin, 1 μM CL316243, 2 μM FCCP, and a mix of 5 μM antimycin A and 5 μM rotenone. Oxygen consumption rates were normalised to the protein content of each well.

### 2.9 Oil Red O staining and quantification

Differentiated brown adipocytes were stained for lipid accumulation using Oil Red O and quantification of Oil Red O accumulation was quantified by spectrophotometry using standard methods. Cells were fixed with 10% formalin for 45 min, incubated with 60% isopropanol for 5 min, and dried at room temperature. Cells were stained with Oil Red O (Sigma–Aldrich, MO, USA) for 10 min and washed with ddH_2_O. The staining was eluted by incubating with 100% isopropanol for 10 min. The absorbance of the eluate was measured in duplicates at 540 nm with an EnSpire plate reader (Perkin Elmer, MA, USA).

### 2.10 Protein isolation from cultured adipocytes

Cells were lysed in IP buffer (25 mM HEPES, pH 7.5, 25 mM Tris–HCl, pH 7.5, 150 mM NaCl, 10 mM EDTA, 0.1% Tween® – 20, 0.5% NP-40, 10 mM beta-glycerolphosphate) containing freshly added inhibitors (1 mM Na_3_VO_4_, 1 mM NaF, 10 μg/ml leupeptin, 10 g/ml aprotinin, 1 mM PMSF, 1x complete protease inhibitor cocktail) by applying at least three freeze and thaw cycles. Samples were centrifuged at 4 °C and 7,500 g for 15 min to obtain cell lysates. Protein concentrations were measured using the Bradford method (Bio-Rad, Protein Assay Dye Reagent Concentrate).

### 2.11 Evaluation of mitochondrial electron transport chain activity

To evaluate mitochondrial electron transport chain activity, the redox dye TTC (2,3,5-Triphenyltetrazolium) was used as previously described [29]. Freshly isolated adipose tissue depots were cut into small pieces (∼0.3 × 0.3 mm) and incubated at 37 °C in 1% TTC in PBS for 15 min. Formazan dye was extracted by incubation in isopropanol at 25 °C overnight. The absorbance of extracts was measured at 485 nm and normalised to tissue weight.

### 2.12 *In silico* analysis for STAT5 binding sites

Sequences of the promoter regions of genes of interest were retrieved using the Ensembl Genome Browser and analysed for the core consensus sequence *TTCN*_*3*_*GAA* for high affinity STAT5 binding until 5000 bp upstream of their transcription start site.

### 2.13 Statistical analyses

Results are presented as mean ± standard error of the mean (SEM). Statistical analyses were performed either by two-tailed Student's t-test or Wilcoxon rank-sum test for comparison of two groups, and one-way ANOVA followed by Tukey's, Dunn's, or Bonferroni's post-hoc tests for multiple comparisons. Cold tolerance tests and bioenergetic profiling of primary brown adipocytes were analysed with repeated measures using two-way ANOVA followed by Bonferroni's post-hoc tests. Data were considered statistically significant: ∗*p* < 0.05; ∗∗*p* < 0.01; ∗∗∗*p* < 0.001.

## 3 Results

### 3.1 Changes in BAT morphology and global gene expression changes upon adipocyte STAT5 deficiency

**Figure 1:**
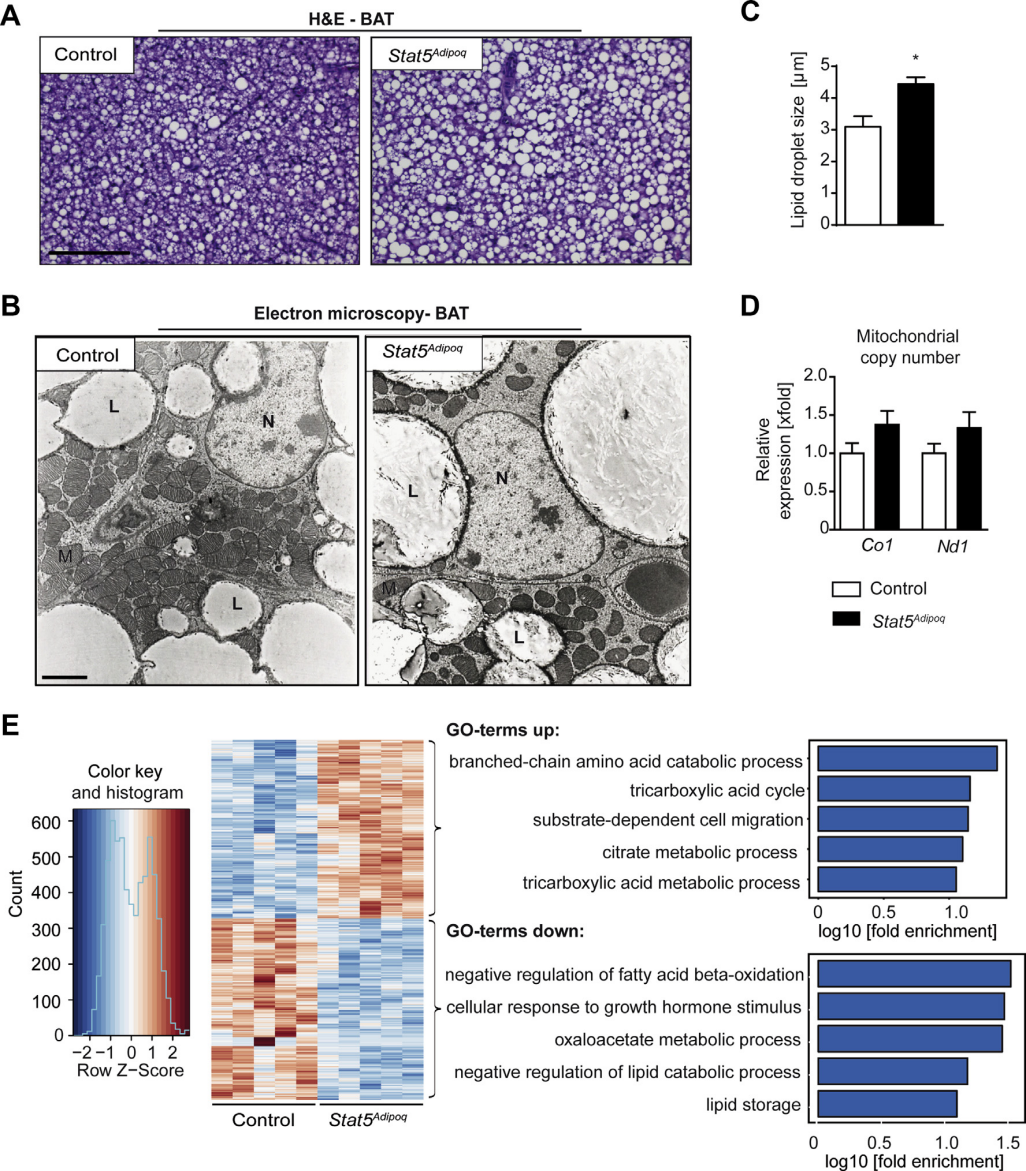
**Impact of STAT5 deficiency on BAT morphology and gene expression**. **A**, Representative haematoxylin and eosin (H&E) staining of paraffin embedded BAT sections (n ≥ 4). Scale bar: 100 μm. **B**, Representative transmission electron microscopy (ELMI) pictures (4000x) of BAT (n = 3/group). Scale bar: 2 μm (L: lipid droplet; M: mitochondria; N: nucleus). **C**, Average lipid droplet diameter was quantified from four ELMI images per sample using Image J (n = 3/group). ∗*p* < 0.05. **D**, Mitochondrial DNA content of BAT. Ct values were normalised to the single copy genomic gene *Ndufv1* (n ≥ 5/group). *Co1*: cytochrome c oxidase subunit 1, *Nd1*: NADH dehydrogenase subunit 1, *Ndufv1*: NADH:ubiquinoneoxidoreductase core subunit V1. **E**, Heat map of differentially expressed genes in BAT obtained from RNA-Seq analysis with corresponding GO analysis for biological processes (n = 5/group).The GO terms displayed have a false discovery rate < 0.05, GO term set size > 5 and fold enrichment over expected > 10. Hierarchically connected, largely redundant GO terms are represented by the smallest set.

To explore the impact of adipocyte STAT5 deficiency on the functionality of BAT, we analysed male *Stat5*^*Adipoq*^ mice [19] in comparison with controls at 2 months of age. While BAT weight was largely unaffected in *Stat5*^*Adipoq*^ mice [19], histology of BAT revealed an increase in lipid droplet size upon STAT5 deficiency (Figure 1A). Analysis of BAT using electron microscopy further supported that lipid droplets were increased in STAT5-deficient brown adipocytes, while we did not observe significant changes in the size or shape of mitochondria (Figure 1B,C). Additionally, mitochondrial DNA copy number was unchanged in BAT of *Stat5*^*Adipoq*^ mice (Figure 1D). Activation status and expression of other STATs such as STAT1 and STAT3 were similar between the genotypes (Suppl. Figure 1A,B). Furthermore, mRNA levels of *Stat1*, *Jak1*, *Jak2* and *Jak3* were unchanged (Suppl. Fig. 1C). To obtain an overview of transcriptional changes occurring in BAT upon adipocyte *Stat5* deletion, we performed RNA-sequencing (RNA-Seq) to compare the gene expression profiles of BAT from *Stat5*^*Adipoq*^ vs. control mice (Figure 1E). Overall, 658 genes were differentially expressed (332 down-regulated, 326 up-regulated) in BAT when STAT5 is lacking. Gene ontology (GO) analysis for down-regulated genes in *Stat5*^*Adipoq*^ BAT revealed an enrichment for pathways linked to lipid metabolism including lipid storage, negative regulation of lipid catabolic processes and negative regulation of fatty acid (FA) β-oxidation. Up-regulated genes were enriched for factors involved in branched-chain amino acid catabolic processes and the tricarboxylic acid (TCA) cycle (Figure 1E). Overall, these data indicate a change in pathways linked to substrate metabolism in BAT in the absence of adipocyte STAT5.

### 3.2 Temperature maintenance upon acute cold exposure is impaired in *Stat5*^*Adipoq*^ mice

**Figure 2:**
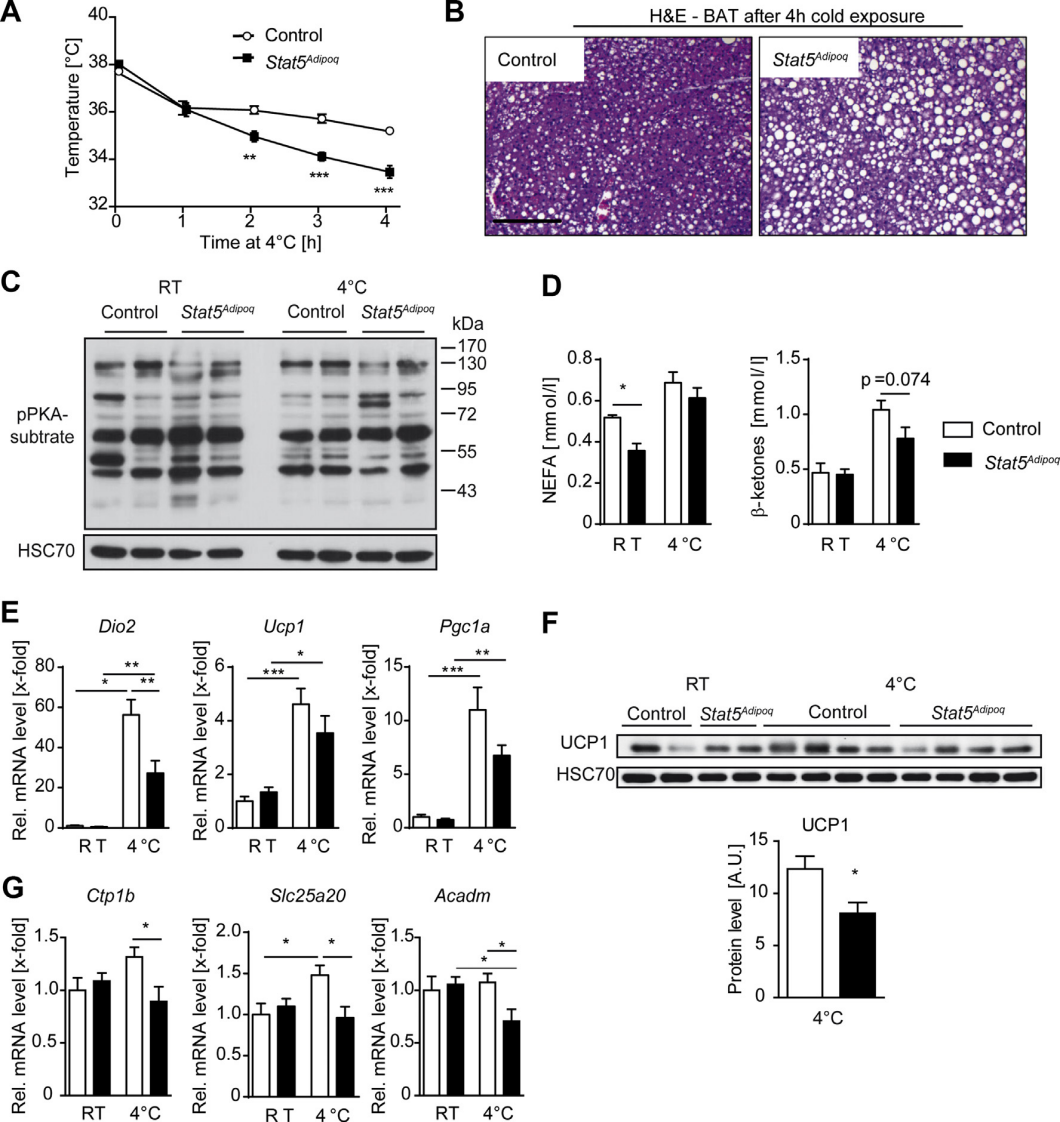
***Stat5*^*Adipoq*^ mice show impaired temperature maintenance during acute exposure to cold**. **A**, Body temperature during acute exposure to cold (n = 6/group). **B**, Representative H&E staining of paraffin embedded BAT sections of mice after cold exposure. Scale bar: 100 μm. **C**, Western blot of PKA activity in BAT lysates of mice maintained at room temperature (RT) and after cold exposure (4 °C). HSC70 was used as loading control. **D**, Blood and plasma metabolite concentrations from mice maintained at RT and after cold exposure (n ≥ 3/group). **E**, Relative mRNA expression of genes involved in thermogenesis in BAT assessed using qPCR. Ct values were normalised to *Gapdh* mRNA levels (n ≥ 5/group, except for *Dio2 Stat5*^*Adipoq*^ mice after exposure to cold n = 4). **F**, Western blot of UCP1 protein levels in BAT of mice maintained at RT and after exposure to cold. Quantification of UCP1 protein levels upon cold exposure was performed using Image J (n = 4/group). **G**, Relative mRNA expression of genes involved in fatty acid oxidation in BAT. Ct values were normalised to *Gapdh* mRNA levels (n ≥ 5/group). ∗*p* < 0.05, ∗∗*p* < 0.01, ∗∗∗*p* < 0.001. *Ucp1*: Uncoupling protein 1, *Dio2*: Deiodinase, iodothyronine Type II, *Pgc1a*: Peroxisome proliferator-activated receptor gamma coactivator 1-alpha, *Cpt1b*: Carnitinepalmitoyltransferase 1B, *Slc25a20*: Solute carrier family 25 (carnitine/acylcarnitinetranslocase), member 20, *Acadm*: Medium-chain specific acyl-CoA dehydrogenase.

To evaluate the role of adipose STAT5 in temperature maintenance, we subjected mice to acute cold exposure. While there was no difference in body temperature between the genotypes when housed at room temperature, body temperature of *Stat5*^*Adipoq*^ mice was significantly reduced compared with controls after only 2 h of exposure to cold (4 °C), indicating impaired temperature maintenance in the absence of STAT5 (Figure 2A). Thermogenesis in BAT requires β-adrenergic activation of lipid mobilisation, FA β-oxidation, and induction of mitochondrial uncoupling [3]. Because *Stat5*^*Adipoq*^ mice showed increased sensitivity to cold stress, we investigated alterations in these processes. Histologic analysis revealed that lipid stores showed a less pronounced decrease in BAT of *Stat5*^*Adipoq*^ mice compared with controls, suggesting inefficient mobilisation or usage of lipids (Figure 2B). Interestingly, we did not observe any induction in HSL activation or PKA activity in BAT of cold-exposed control and *Stat5*^*Adipoq*^ mice (Figure 2C and Suppl. Figure 2A–C). To fuel thermogenesis, BAT utilizes lipids that are stored within BAT depots as well as NEFA that are provided by WAT. Circulating NEFA levels remained slightly, but not significantly, lower in cold-exposed *Stat5*^*Adipoq*^ mice compared with controls (Figure 2D), indicating that cold-induced lipid mobilisation from WAT *per se* was not impaired in *Stat5*^*Adipoq*^ mice. Additionally, blood β-ketones, an indicator of lipid mobilisation and redistribution, tended to be lower in cold-exposed *Stat5*^*Adipoq*^ mice (Figure 2D).

We analysed mRNA expression of genes that are relevant for the induction of the thermogenic program in BAT to explore molecular changes that underlie the impairment in temperature maintenance of *Stat5*^*Adipoq*^ mice. The expression of cold-induced gene *Dio2* (encoding deiodinase, iodothyronine type II) was significantly attenuated in cold-exposed *Stat5*^*Adipoq*^ mice, while *Pgc1a* (peroxisome proliferator-activated receptor gamma coactivator 1-alpha) expression tended to be lower (Figure 2E). Furthermore, both mRNA and protein expression analysis showed that UCP1 was not efficiently expressed in STAT5-deficient BAT after exposure to cold (Figure 2E,F). Additionally, genes that encode proteins involved in FA β-oxidation such as *Cpt1b* (carnitinepalmitoyltransferase 1B), *Slc25a20* (carnitine-acylcarnitinetranslocase), and *Acadm* (acyl-CoA dehydrogenase medium chain) showed a collective reduction in expression in *Stat5*^*Adipoq*^ mice upon exposure to cold (Figure 2G).

Collectively, these data indicate that adipose STAT5 deficiency impairs acute cold-induced temperature maintenance, which is associated with reduced lipid usage and expression of UCP1.

### 3.3 Stimulated lipolysis is impaired upon STAT5 deficiency in BAT

**Figure 3:**
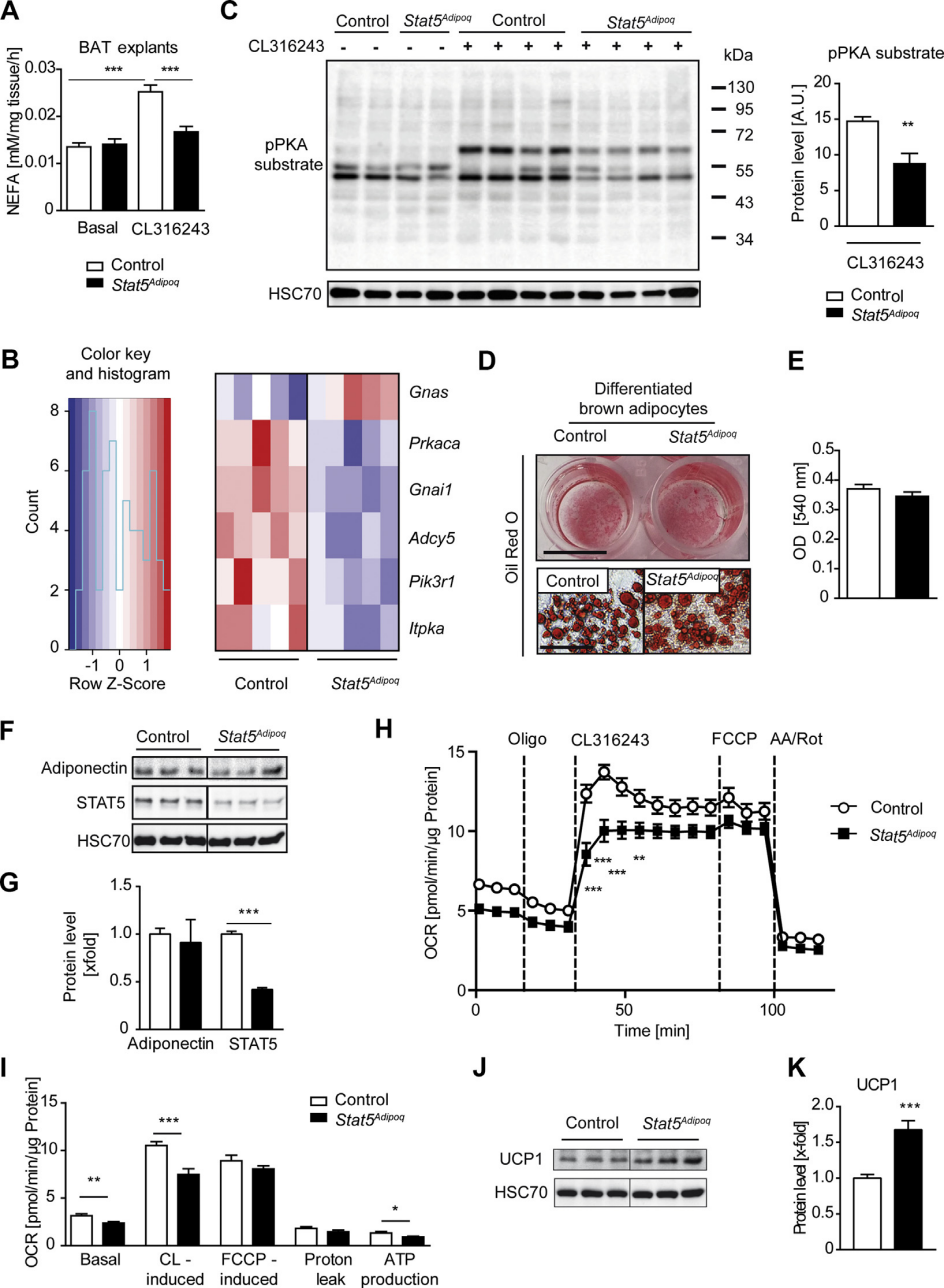
**β_3_-adrenergic stimulation is blunted in STAT5-deficient BAT and associates with diminished PKA activity**. **A**, Basal and CL316243-stimulated lipolysis represented by NEFA release from BAT explants (n ≥ 9 explants from 3 mice/group). **B**, Heatmap of genes involved in β-adrenergic signalling that show significant (*p* < *0.05*) changes in expression in BAT of *Stat5*^*Adipoq*^ mice assessed by RNA-Seq. **C**, Western blot for pPKA substrate of lysates from BAT explants used in A. The signal for CL316243-induced pPKA substrate was quantified using Image J (n = 4/group). **D**, Representative Oil Red O staining of primary differentiated brown adipocytes at low magnification (upper panel, scale bar: 0.5 cm) and high magnification (lower panel, scale bar: 50 μm). Quantification (**E**) of Oil Red O content was performed by measuring the absorbance of the eluted staining (n = 5/genotype). **F**, Western blot analysis for adiponectin and STAT5 in lysates of primary differentiated brown adipocytes. HSC70 was used as loading control. Samples were loaded on the same gel but not consecutively as indicated. Quantification (**G**) was performed using ImageJ (n = 3/genotype). **H**, Bioenergetic profile of primary differentiated brown adipocytes of control and *Stat5*^*Adipoq*^ mice shown as a representative curve. Baseline oxygen consumptions rates (OCR) and OCRs in the presence of oligomycin (Oligo), CL316243, FCCP and antimycin A (AA)/rotenone (Rot) were analysed using an XFe96 Seahorse Bioscience analyser (n = 16 wells/group). **I**, Respiratory capacities of primary brown adipocytes are shown from a representative experiment (n = 16 wells/group) from three experiments. **J**, Western blot for UCP1 in lysates of primary differentiated brown adipocytes. HSC70 serves as loading control. Samples were loaded on the same gel but not consecutively as indicated. Quantification (**K**) was performed using Image J (n = 6/genotype). ∗*p* < 0.05, ∗∗*p* < 0.01, ∗∗∗*p* < 0.001.

Because our data indicated a diminished mobilisation or usage of BAT lipid stores during exposure to cold, we evaluated lipolysis *ex vivo* using BAT explants. While basal lipolysis was similar between control and *Stat5*^*Adipoq*^ BAT, stimulation with the selective β_3_-adrenergic agonist CL316243 revealed that the lipolytic response was blunted in STAT5-deficient BAT (Figure 3A). Interestingly, stimulation of BAT explants with GH did not result in an increase in the lipolytic rate in either genotype, suggesting that GH does not have an acute lipolytic effect in BAT (Suppl. Fig. 3A). To explore molecular changes that may underlie the blunted lipolytic response upon β_3_-adrenergic stimulation, we tested whether STAT5 deficiency in BAT interferes with the expression of genes involved in the β_3_-adrengergic pathway. Analysis of RNA-Seq data showed that expression of *Adrb3* (β_3_-adrenergic receptor) as well as most adenylyl cyclases were unchanged between the genotypes (Suppl. Fig. 3B). However, *Stat5* deletion led to significantly reduced expression of *Adcy5* (adenylate cyclase 5), *Gnai1* (G protein subunit alpha I1), *Itpka* (inositol-trisphosphate 3-kinase A), *Pik3r1* (phosphoinositide-3-kinase regulatory subunit 1), and *Prkaca* (protein kinase A catalytic subunit alpha), while expression of *Gnas* (guanine nucleotide binding protein, alpha stimulating activity polypeptide) was increased (Figure 3B) indicating that these alterations in gene expression might impinge on the β-adrenergic responsiveness of the tissue. *In silico* analysis of promoter regions of these genes revealed six high affinity STAT5 binding sites according to the consensus site determination [30] in *Adcy5*, *Itpka* and *Pik3r1* (Suppl. Fig. 3C), suggesting that STAT5 could potentially directly influence their transcription. We evaluated PKA activity in stimulated BAT explants to verify functionally whether β_3_-adrenergic signalling is impaired. In line with the attenuated lipolytic response, the phosphorylation of PKA substrates following CL316243 stimulation was significantly reduced in STAT5-deficient BAT (Figure 3C).

Collectively, these data demonstrate that loss of STAT5 interferes with β_3_-adrenergic responsiveness and concomitant lipolysis in BAT.

### 3.4 Basal and UCP1 dependent respiration is diminished in primary differentiated brown adipocytes of *Stat5*^*Adipoq*^ mice

To further define the importance of STAT5 for brown adipocyte functionality, we differentiated primary brown adipocytes from control and *Stat5*^*Adipoq*^ mice. The differentiation capability was similar between the genotypes, as assessed by Oil Red O staining (Figure 3D,E) and expression of adiponectin (Figure 3F,G). Additionally, STAT5 expression was significantly reduced in differentiated primary brown adipocytes from *Stat5*^*Adipoq*^ mice compared with controls (Figure 3F,G). We then determined the bioenergetic profile of primary differentiated brown adipocytes from control and *Stat5*^*Adipoq*^ mice because mitochondrial respiration is an indicator of the metabolic activity of BAT (Figure 3H). After evaluating basal respiration, the ATP synthase inhibitor oligomycin was added to discriminate between oxygen consumption rates coming from ATP synthesis or from proton leak. We used the β_3_-adrenergic agonist CL316243 [28] and general uncoupling was induced by adding carbonyl cyanide 4-(trifluoromethoxy) phenylhydrazone (FCCP) to determine UCP1 dependent respiration. A mix of antimycin A and rotenone served to determine non-mitochondrial oxygen consumption. Notably, primary differentiated brown adipocytes from *Stat5*^*Adipoq*^ mice showed a reduction in basal respiration as well as CL316243-induced respiration, while FCCP–induced respiration appeared similar between the genotypes (Figure 3I). Analysis of various genes in the TCA cycle and ETC revealed an increase in mRNA levels of *Cs* (citrate synthase) and *Aco2* (aconitase 2) (Suppl. Figure 3D,E). In contrast to the *in vivo* findings, UCP1 expression was increased in primary brown adipocytes from *Stat5*^*Adipoq*^ mice (Figure 3J,K) suggesting the reduction in respiration observed was not because of a defect in UCP1 expression, but rather because of a defect in its activation and/or substrate availability.

In conjunction, these results allow us to conclude that STAT5 deficiency interferes with the respiratory capacity of brown adipocytes.

### 3.5 Adipocyte STAT5 deficiency attenuates the remodelling capacity of WAT and inhibits the induction of mitochondrial respiration upon chronic β_3_-adrenergic stimulation

**Figure 4:**
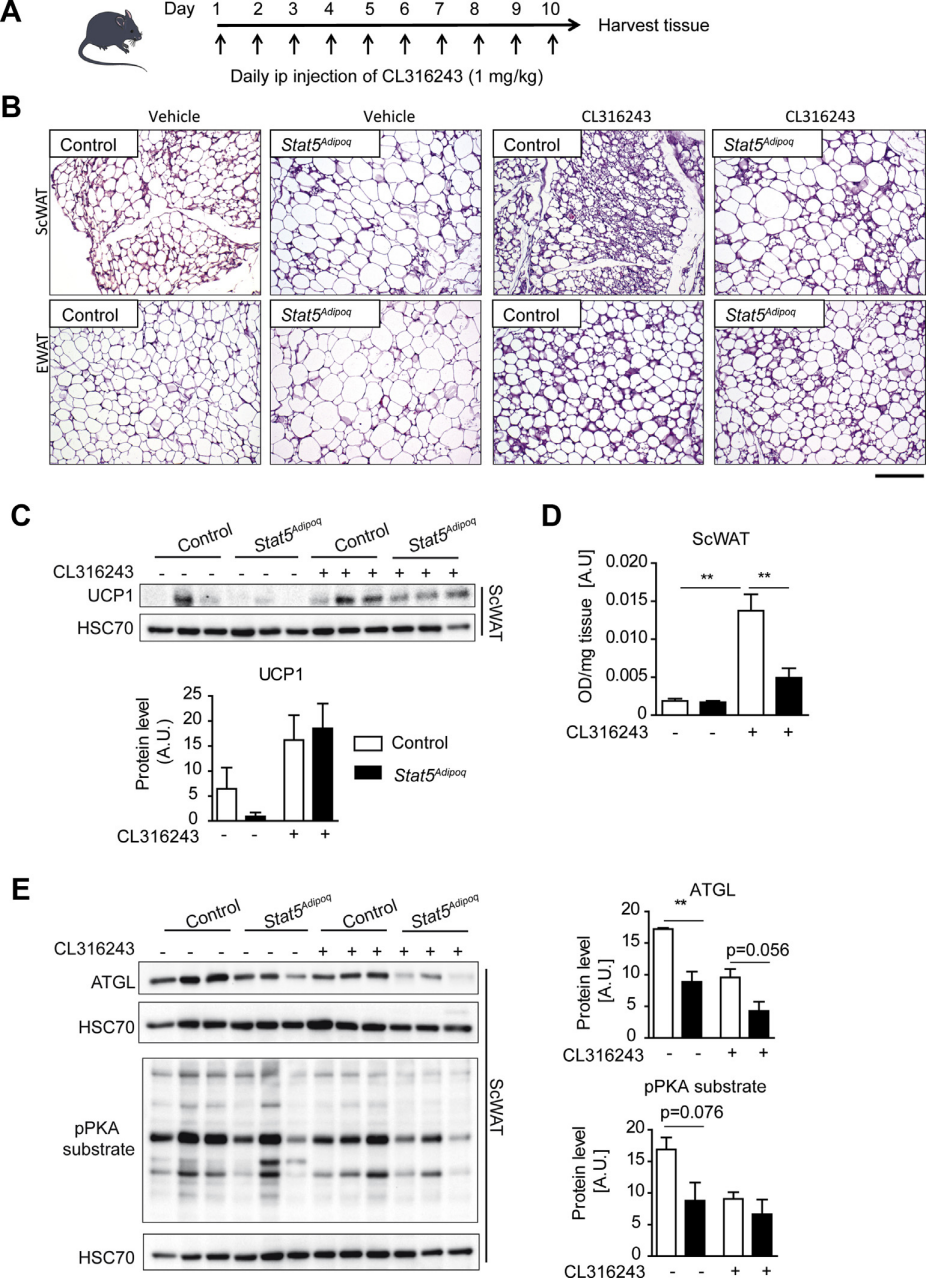
**STAT5 is required for tissue remodelling and the induction of mitochondrial respiration in ScWAT after chronic β-adrenergic treatment**. **A**, Experimental set up of chronic β-adrenergic stimulation using CL316243. Samples were collected on day 11. **B**, Representative H&E staining of paraffin embedded sections of ScWAT and EWAT from vehicle and CL316243 treated mice. Scale bar: 100 μm. **C**, Western blot of UCP1 protein levels in ScWAT after mock and CL316243 treatment. Quantification was performed using Image J. HSC70 was used as loading control (n = 3/group). **D**, Mitochondrial respiration in ScWAT was determined by reduction of the electron acceptor dye TTC (-CL316243 n = 3/group; +CL316243 n = 6/group). **E**, Western blot for pPKA substrate and for ATGL in ScWAT after vehicle and CL316243 treatment. Quantification was performed using Image J. HSC70 was used as loading control (n = 3/group).

Chronic exposure to cold or β_3_-adrenergic stimulation results in WAT remodelling and the appearance of beige adipocytes within WAT depots [31]. To examine whether STAT5 plays a role in WAT browning, we mimicked chronic cold exposure by treating mice with the selective β_3_-adrenergic agonist CL316243 for 10 days (Figure 4A). While CL316243 treatment did not have an impact on epididymal WAT (EWAT) depot size, a significant reduction in subcutaneous WAT (ScWAT) mass was observed in response to CL316243 treatment in *Stat5*^*Adipoq*^ mice (Suppl. Fig. 4A). Additionally, an increase in BAT weight was observed in the control mice upon treatment (Suppl. Fig. 4A). Histological analysis of CL316243-treated control ScWAT displayed extensive tissue remodelling. As expected, treatment with the β_3_-adrenergic agonist promoted the appearance of multilocular adipocytes within ScWAT depots of the control mice, which is indicative of WAT browning (Figure 4B). Although multilocular adipocytes were observed in ScWAT depots of *Stat5*^*Adipoq*^mice, the tissue remodelling effect of chronic β_3_-adrenergic stimulation was less pronounced (Figure 4B). In EWAT, the tissue remodelling effect upon CL316243 treatment was not as drastic as in ScWAT but appeared to be similar between the genotypes. These data show that adipocyte STAT5 is required for ScWAT remodelling upon chronic β_3_-adrenerigc stimulation.

As browning of WAT is usually associated with the expression of thermogenic factors [10], we next assessed how *Stat5* deletion affects the expression of UCP1. Surprisingly, although tissue remodelling was highly diminished in STAT5-deficient ScWAT, Western blot analysis revealed a similar induction of UCP1 proteins levels (Figure 4C). We then analysed mRNA levels of genes involved in the β_3_-adrengergic pathway that were differentially expressed in BAT of *Stat5*^*Adipoq*^ mice (Figure 3B) and found that only *Pik3r1* was significantly reduced in STAT5-deficient ScWAT after CL316243 treatment (Suppl. Fig. 4B). To further analyse whether STAT5 was required for the CL316243-induced effects on mitochondrial respiration, we analysed the activity of the electron transport chain *in situ* using the redox dye TTC. Although UCP1 expression was similar between the genotypes after chronic β-adrenergic treatment in ScWAT, STAT5 deficiency highly diminished the CL316243-induced upregulation of electron transport activity (Figure 4D). As substrate availability through lipolysis is essential for the induction of the mitochondrial respiration in WAT, we further evaluated the expression of key factors involved in lipolysis. In accordance with previous data [19], PKA activity was not drastically different in ScWAT between the genotypes, while adipose triglyceride lipase (ATGL) expression was diminished in *Stat5*^*Adipoq*^ ScWAT (Figure 4E), which suggests that the diminished substrate availability might be responsible for the reduction in mitochondrial activity in ScWAT of *Stat5*^*Adipoq*^ mice.

## 4 Discussion

We have shown that adipose STAT5 deficiency impaired temperature maintenance upon acute exposure to cold, which coincided with a diminished usage of BAT lipid stores and reduced UCP1 expression. Interestingly, the lipolytic response upon β_3_-adrenergic stimulation was blunted in STAT5-deficient BAT, which was partly attributed to diminished PKA activity. Accordingly, the respiratory capacity, as a readout for mitochondrial function, was reduced in primary differentiated adipocytes from *Stat5*^*Adipoq*^ mice. Moreover, STAT5 deficiency diminished brown remodelling of ScWAT upon chronic β_3_-adrenergic stimulation. This, however, was not associated with an impaired induction of UCP1 expression, but with a diminished respiratory activity.

Adipocyte STAT5-deficiency reduced the ability of *Stat5*^*Adipoq*^ mice to maintain body temperature during acute cold stress, indicating an impairment in adaptive thermogenesis. As reported previously, basal WAT lipolysis was diminished in *Stat5*^*Adipoq*^ mice resulting in lower circulating NEFA levels [19]. In addition to their usage in mitochondrial FA β-oxidation to provide substrates for uncoupling, free FAs also directly increase UCP1-mediated proton transport activity [32] and are therefore indispensable for thermogenesis [4]. In fact, various transgenic mouse models with defects in lipid mobilisation were shown to be sensitive to cold stress [[33], [34], [35]]. Furthermore, recent studies suggest that WAT lipolysis is potentially more important for thermogenesis than BAT lipolysis; defective lipid mobilisation in BAT, at least mediated via the major lipase ATGL and its coactivator CGI-58, does not impair temperature maintenance [36,37]. Interestingly, exposure to cold in our study increased circulating NEFA levels almost to the same level in both genotypes, arguing against disturbances in WAT-derived NEFA flux during cold exposure upon STAT5 deficiency. However, the amount of lipid droplets was increased in BAT of *Stat5*^*Adipoq*^ mice, suggesting a diminished lipid breakdown in BAT, while UCP1 expression was reduced after exposure to cold. Additionally, circulating β-ketone levels tended to be lower in cold-exposed *Stat5*^*Adipoq*^ mice, potentially contributing to the increased sensitivity to cold exposure [38]. Hence, the aberrant cold response of *Stat5*^*Adipoq*^ mice is likely a combination of impaired BAT lipid mobilisation and usage as well as reduced expression of UCP1. In accordance with our data, a recent study showed that adipose JAK2, the prototypical upstream kinase of STAT5, is required for diet- and cold-induced thermogenesis in mice, and JAK2 deficiency was also associated with diminished UCP1 expression [15]. Although some of the effects we find in our study might be attributed to secondary effects mediated by STAT5-deficient WAT, our data suggest that STAT5 deficiency also affects BAT functionality in a cell-intrinsic way. The induction of lipolysis via the β_3_-adrenergic agonist CL316243 was blunted in BAT explants of *Stat5*^*Adipoq*^ mice, which was at least partly attributed to diminished activation of PKA. These results contrast with the lipolytic response seen in WAT explants of *Stat5*^*Adipoq*^ mice, in which basal lipolysis is diminished, but the response to β-adrenergic stimulation is intact [19]. Our results thereby suggest that STAT5 fulfils different functions in WAT and BAT and indicate that STAT5 is required for β_3_-adrenergic responsiveness of BAT.

Furthermore, primary differentiated brown adipocytes derived from *Stat5*^*Adipoq*^ BAT showed reduced CL316243-induced respiration suggesting a defect in UCP1 activation, likely a result of the diminished induction of intracellular fatty acid levels. In accordance, it was shown that lipolysis is required for the induction of respiration in primary brown adipocytes after β-adrenergic stimulation [28].

The canonical upstream signalling cascades, members of the JAK-STAT pathway were likewise also shown to be activated by G-protein-coupled receptors such as angiotensin II type 1 receptor [39,40], and CCR2 and CXCR4 chemokine receptors [41,42]. However, it has not been shown that STAT5 can be activated by β_3_-adrenergic agonists in BAT. RNA-Seq profiling showed a significant down regulation of several genes of the β-adrenergic signalling cascade in BAT of *Stat5*^*Adipoq*^ mice, including those encoding PKA and ADCY5. Interestingly, the stimulatory G-protein alpha subunit (*Gnas*) was up-regulated, while the inhibitory G-protein alpha I1 subunit (*Gnai1*) was down-regulated, which might be a compensatory mechanism to normalise the β_3_-adrenergic response in STAT5-deficient BAT. We also identified multiple putative high affinity STAT5 binding sites in *Adcy5*, *Itpka* and *Pik3r1* promoter regions hinting at a potential direct transcriptional regulation of these genes by STAT5. Although these data provide evidence that the blunted CL316243 response might partly involve impaired transcriptional regulation by STAT5, molecular and functional studies will be necessary to clarify this further. Along these lines, an important aspect to be addressed in future studies will be the identification of the upstream mediator(s) of STAT5 in BAT, which are responsible for the phenotypes observed.

Chronic β_3_-adrenergic activation induces extensive WAT remodelling, an increase in UCP1- expressing adipocytes, and the induction of the thermogenic program [5]. In this regard, it is anticipated that ScWAT is more prone to browning than EWAT [43]. STAT5 deficiency blocked ScWAT remodelling following chronic β_3_-adrenergic treatment, and this finding is supported by a similar observation in systemic GH receptor-truncated mice [44]. Interestingly, the induction of UCP1 expression was not affected in *Stat5*^*Adipoq*^ ScWAT, which suggests that UCP1 expression in ScWAT might be independent of extensive tissue remodelling in *Stat5*^*Adipoq*^ mice. Importantly, the increase in mitochondrial respiration promoted by CL316243 treatment was blunted in the ScWAT of *Stat5^Adipoq^* mice. In accordance with previous reports [19], STAT5 deficiency diminished the expression of the first lipase required for triglyceride breakdown, ATGL, in ScWAT. Because the induction of UCP1 protein expression was similar to those in controls, it is likely that the defect in mitochondrial respiration depends on the substrate supply. In support of this concept, inducible deletion of ATGL in mice eliminated the CL316243-mediated upregulation of electron chain activity in various adipose tissue depots [45].

In summary, we have shown for the first time that STAT5 is crucial for the β-adrenergic responsiveness of BAT. Thus, STAT5 represents a key factor that mediates the mobilisation of free FAs from BAT lipid stores to fuel thermogenesis and mitochondrial respiration. Along these lines, STAT5 determines the capacity of β-adrenergic-induced white fat remodelling and mitochondrial respiration. These novel insights into the requirement of STAT5 for the physiology of thermogenic fat and its role in lipid metabolism can pave the way for future studies addressing how its inactivation affects outcomes in metabolic diseases associated with obesity.

## Funding

RM and DK were supported by the 10.13039/501100002428Austrian Science Fund (FWF) [SFB-F04707, SFB-F06105, SFB-F06107, under the frame of ERA-NET (I 4157-B)] as well as generously supported by a private donation from Liechtenstein. RM, MH, and KS were also funded by the EU Transcan-2 consortium ERANET-PLL via the 10.13039/501100002428FWF and the 10.13039/100012893DLR. This project received additional funding from the 10.13039/100010663European Research Council (ERC) under the European Union's Horizon 2020 research and innovation programme (grant agreement n° 636855 to FG).

## Author contribution

Most experiments were performed by DK and KS with support from KMM. Experiments were planned by DK and KMM with support from RM. FR performed bioinformatic data analysis. AR and EP supported Seahorse experiments. LK supported electron microscopy imaging. RM supervised the study and acquired most of the required funding for the study. DK wrote the manuscript with support from KMM, KS, and RM. RM, FG, MH, and FR critically revised the manuscript. RM, FG, and MH also financed the study. All authors read, corrected, and approved the final version of the manuscript. RM is responsible for the integrity of the study.
