# Supplementary material for: STAT5 is required for lipid breakdown and beta-adrenergic responsiveness of brown adipose tissue
Source: Mol Metab. 2020 May 28;40:101026. doi: 10.1016/j.molmet.2020.101026 (PMC7322099; doi:10.1016/j.molmet.2020.101026)
Supplement: Multimedia component 1 [file mmc1.pdf]

Supplementary information

Isolation and differentiation of primary brown adipocytes.

Cells of the stroma vascular fraction (SVF) were isolated from BAT of 4-5 week old mice. Briefly, freshly isolated BAT was minced into small pieces followed by digestion in serum free DMEM/F-12 supplemented with 1 mg/ml collagenase II, 75 U/ml DNAse I and 1,5% BSA for 60 min and moderate shaking (140 rpm) at 37°C. Cell suspensions were filtered through a 100 µm cell strainer and centrifuged for 5 min at 200 g. The pellet was washed twice in 5 ml growth medium (DMEM/F-12, 10% FBS, 1% glutamine, 1% penicillin/streptomycin) followed by an incubation in 1 ml erythrocyte lysis buffer (Qiagen) for 5 min. Erythrocyte lysis was stopped by adding growth medium to 12 ml and another round of centrifugation followed at 200 g for 5 minutes . The pellet was resuspended in growth medium and filtered through a 70 µm cell strainer. After the final centrifugation step (200 g, 5 min) cells of the SVF were resuspended in 150 µl growth medium and the cell count was determined. Cells were seeded into a XF96 V3-PS cell culture microplate (Agilent Bioscience) with a seeding density of 2000 cells per well. After cells were confluent, adipocyte differentiation was induced by replacing the growth medium with induction medium (growth medium supplemented with 0.5 mM 3-isobutyl-1-methylxanthine, 125 nM indomethacin, 1 nM 3,3′,5 triiodothyroninesodium salt (T3), 0.86 µM insulin, 1 µM dexamethasone 1 µM rosiglitazone) and the induction medium was exchanged every other day. On day 7, cells were grown in post differentiation medium (growth medium supplemented with 1 nm T3, 0.86 µM insulin and 1 µM rosiglitazone).

Supplementary Table 1: List of oligos

Oligo	Sequence (5´- 3´)
**Genotyping**	
*Cre*_1<br>*Cre*_2	ACCAGCCAGCTATCAACTCG<br>TTACATTGGTCCAGCCACC
**qPCR**	
*Gapdh*_1<br>*Gapdh*_2	AGAAGGTGGTGAAGCAGGCATC<br>CGGCATCGAAGGTGGAAGAGTG
36B4_1<br>*36B4_2*	GCTTCATTGTGGGAGCAGACA<br>CATGGTGTTCTTGCCCATCAG
Ucp1_1<br>*Ucp1_2*	GACGTCCCCTGCCATTTAC<br>CGCAGAAAAGAAGCCACAA
Ppargc1a_1<br>*Ppargc1a_2*	TGAGGACCGCTAGCAAGTTT<br>TGAAGTGGTGTAGCGACCAA
Dio2_1<br>*Dio2_2*	ACACTGGAATTGGGAGCAT<br>ATGCTGACCTCAGAAGGGCT
Ctp1b_1<br>*Ctp1b_2*	GCTGCTTGCACATTTGTGTT<br>TGTCTACCTCCGAAGCAGGA
Scl25a20_1<br>*Scl25a20_2*	AAACTTGGTGGTTGTGTCTGC<br>TTTAAGAACCTCCTGGCTGG
Acadm_1<br>*Acadm_2*	CAACCTTCATCGCCATTTCT<br>GCCCAGAGAGCTCTAGACGA
Stat1_1<br>*Stat1_2*	TGGCGAGAACCTAAGTTTCAGT<br>CCCAAAGGGCAAAAAGAACT
Jak1_1<br>*Jak1_2*	AGTGCAGTATCTCTCCTCTCTG<br>GATTCGGTTCGGAGCGTACC
Jak2_1<br>*Jak2_2*	GGAATGGCCTGCCTTACAATG<br>TGGCTCTATCTGCTTCACAGAAT
Jak3_1<br>*Jak3_2*	ACACCTCTGATCCCTCAGC<br>GCGAATGATAAACAGGCAGGATG
Gnas_1<br>*Gnas_2*	CCCTCTCCGTTAAACCCATT<br>CTCCGTCCAGATTCTCCTTG
Prkaca_1<br>*Prkaca_2*	TTCTCTTCCTGTTCCCACCCT<br>ACTCAGTCTTTCTCAGGGCAC
Gnai1_1<br>*Gnai1_2*	TCGCCAACTTTTCGTGCTTG<br>TCAGATAGTACGCTGCCGAA
Adcy5_1<br>*Adcy5_2*	TGGAGATGGGAATGGACATGAT<br>CACGCGCATGTTCACGTT
Pik3r1_1<br>*Pik3r1_2*	CTGAAGCTGACACGGAGCAGC<br>GACGTGTACGTCGATCATCTC
Itpka_1<br>*Itpka_2*	CTGAAGTACTCGCCCTTCGT<br>TGCTCACACTGACAGAAACG
Cox2_1<br>*Cox2_2*	GCCGACTAAATCAAGCAACAG<br>CTAGGACAATGGGTATAAAGCTAT
Cox4i1_1<br>*Cox4i1_2*	ACCAAGCGAATGCTGGACAT<br>GGCGGAGAAGCCCTGAA
Cox7a_1<br>*Cox7a_2*	CCGACAATGACCTCCCAGTACAC<br>CCAGCCCAAGCAGTATAAGCAGT
Cox8b_1<br>*Cox8b_2*	TATCCTGCGGCTGCTCCAA<br>CGACTATGGCTGAGATCCCCAC
Cs_1<br>*Cs_2*	AGCCAAGAACTCATCCTG<br>TCTTCCCATGTTGCTGCTTGA
Aco1_1<br>*Aco1_2*	GGGGTGTGGGTGGTATTGAA<br>CTTGTCGGAGGTGCTTGGTAAT

Aco2_1<br>*Aco2_2*	GCTGACCCCTCCGACTATAACA<br>ATGACGCACTTCAGAGGCTTTC
Idh1_1<br>*Idh1_2*	ACTCAGTCGCCCAAGGTTATG<br>CCCTTTCTGGTACATGCGGT
Sdha_1<br>*Sdha_2*	AAACAGACCTGCGGCTTTCA<br>AGCATTGATACCTCCCTGTGC
Sdhb_1<br>*Sdhb_2*	GCGGACCTATGGTGTTGGAT<br>GAGCCACAGATGCCTTCTCT
Mdh2_1<br>*Mdh2_2*	CCGCCTGACCCTCTACG<br>CATCCCGTGTCATTCCTGGTT
Mitochondrial copynumberdetermination	
Co1_1<br>*Co1_2*	TGCTAGCCGCAGGCATTAC<br>GGGTGCCCAAAGAATCAGAAC
Nd1_1<br>*Nd1_2*	GAGCCTCAAACTCCAAATACTCAC<br>AAAGGATAATAGCTATGGTTACTTCA
Ndufv1_1<br>*Ndufv1_2*	CTTCCCCACTGGCCTCAAG<br>CCAAAACCCAGTGATCCAGC

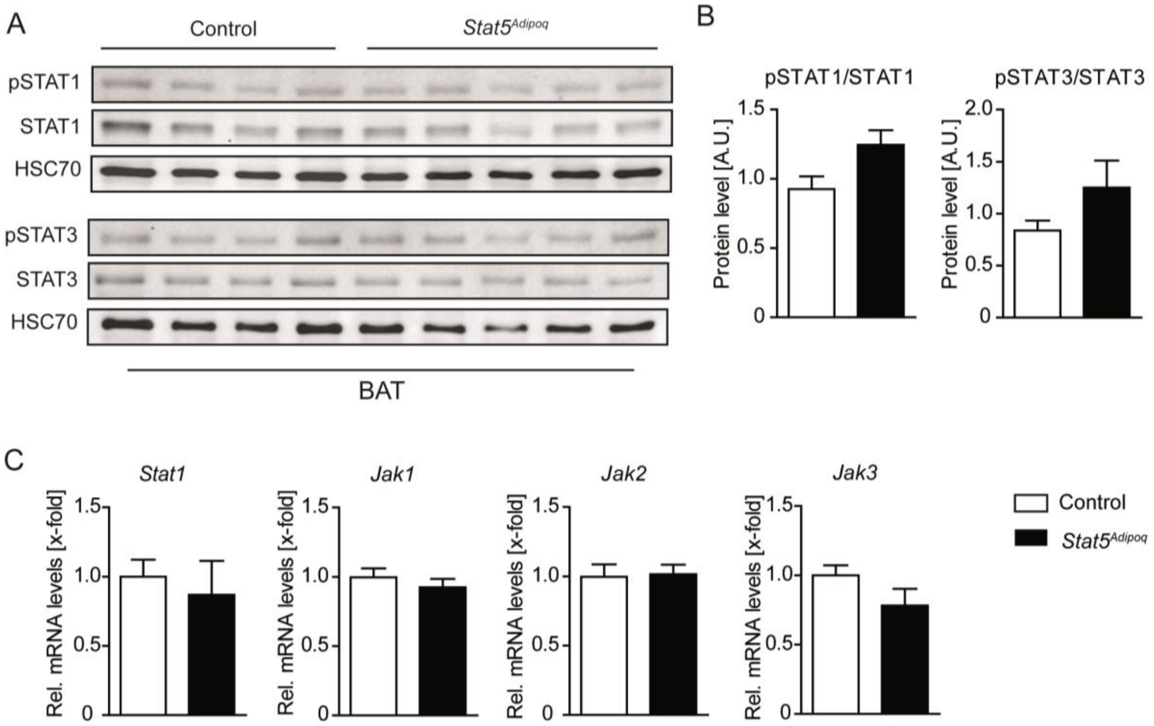

Suppl. Figure 1: **Analysis of members of the JAK-STAT signalling pathway in BAT.A** Western blot for the expression and activation status of STAT1 and STAT3 in BAT lysates. HSC70 was used as loading control. Quantification (**B**) was performed using ImageJ (n=4-5 /group). **C** Relative mRNA expression of *Stat1*, *Jak1, Jak2* and *Jak3*. Ct values were normalised to *Gapdh* mRNA levels (n=7/group).

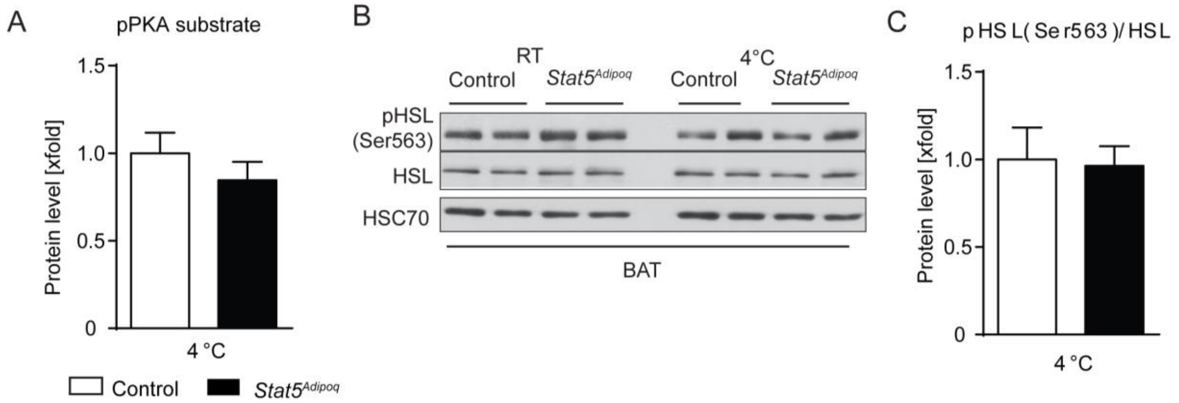

Suppl. Fig. 2: Analysis of hormone sensitive lipase expression in BAT. **A** Quantification of pPKA substrate levels from Fig. 2C upon cold exposure was performed using ImageJ (n=6/group). **B** Activation and expression status of HSL in BAT lysates. HSC70 was used as loading control. **C** Quantification of HSL activation upon cold exposure was performed using ImageJ (n=6/group).

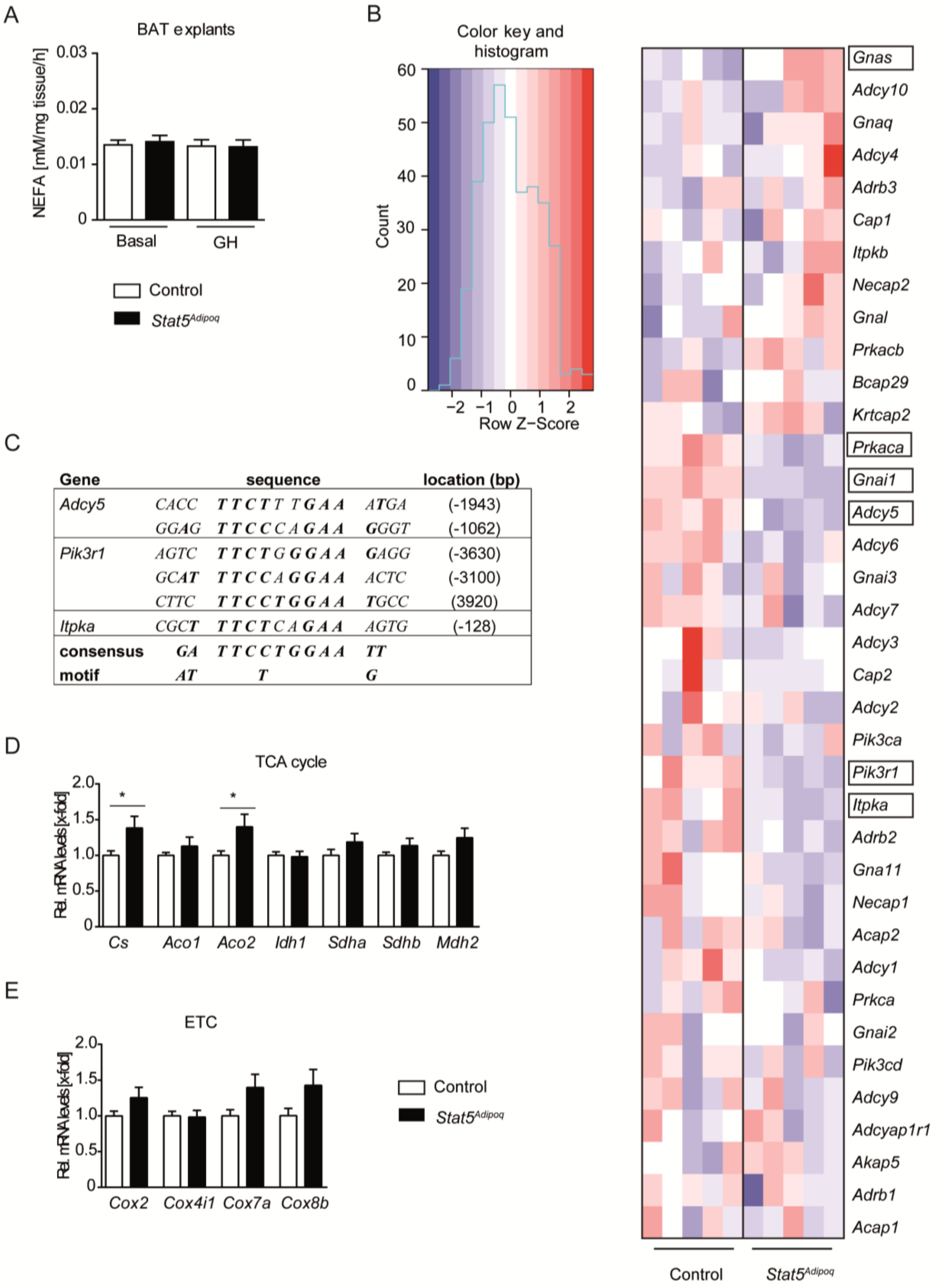

Suppl. Fig. 3: GH does not stimulate acute lipolysis in BAT. **A** Basal and GH stimulated lipolysis represented by NEFA release from BAT explants (n ≥ 8 explants).Values for basal lipolysis are similar to the ones shown in Fig. 3A. **B** Heatmap displaying genes involved in β-adrenergic signalling in BAT as assessed by RNA-Seq. Names of Genes which show significant

changes in gene expression (p<0.05) are displayed in boxes. **C** Putative high affinity STAT5 binding sites (highlighted in bold) according to the consensus sequence were identified in promoter regions of *Adcy5*, *Pik3r* and *Itpka* by performing an *in silico* analysis of the respective promoter region. The number indicates the distance in bp from the transcriptional start site. **D** and **E**: Relative mRNA expression of genes involved in the electron transport chain (ETC) (**D**) and tricarboxylic acid (TCA) cycle (**E**) in differentiated brown adipocytes. Ct values were normalised to *36B4* mRNA levels (n=8/group). *$p < 0.05$. *36B4*: acidic ribosomal phosphoprotein P0, *Cox2*: Cytochrome C oxidase subunit 2, *Cox4i1*: Cytochrome C oxidase Subunit 4 isoform 1, *Cox7a*: Cytochrome C oxidase Subunit 7A1, *Cox8b*: Cytochrome C oxidase Subunit 8B, *Cs:* Citrate synthase, *Aco1*: Aconitase 1, *Aco2*: Aconitase 2, *Idh1*: Isocitrate dehydrogenase (NADP(+)) 1), *Sdha* : Succinate dehydrogenase complex flavoprotein subunit A, *Sdhb:* Succinate dehydrogenasecomplex iron sulfur subunit B, *Mdh2*: Malate dehydrogenase 2

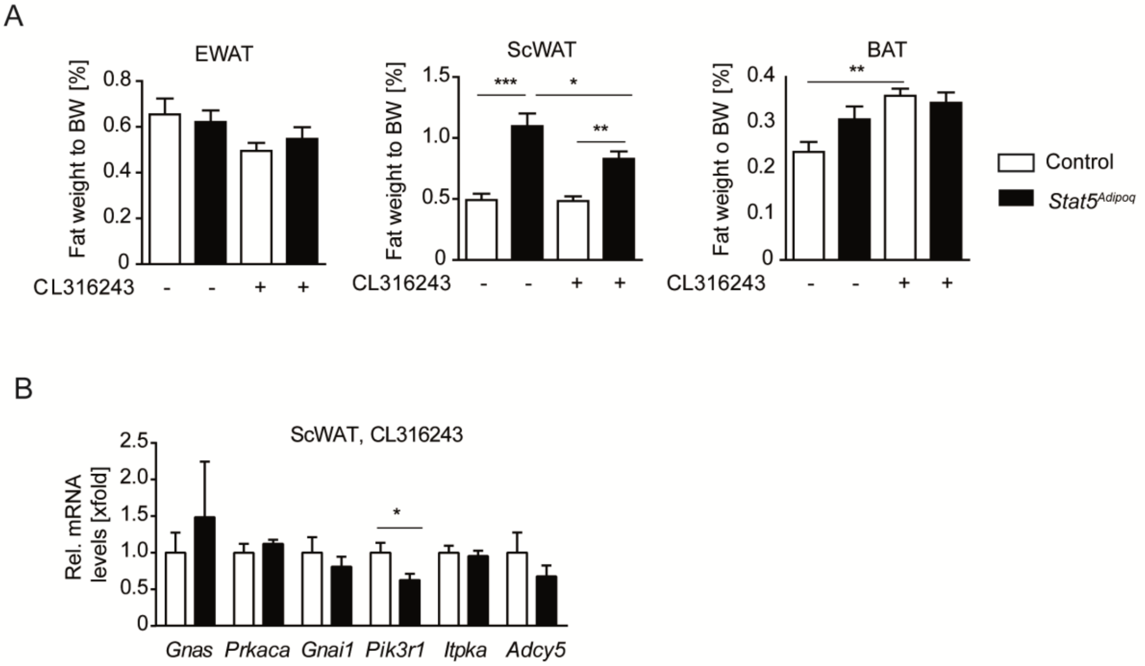

Suppl. Fig. 4: Effects of chronic β3-adrenergic stimulation on tissue weight and gene expression. A Adipose tissue weight in relation to body weight (BW) from vehicle- and CL316243-treated mice (n=6/group). **B** Relative mRNA expression of genes involved in β-adrenergic signalling pathway in ScWAT after chronic CL316243 treatment. Ct values were normalized to *Gapdh* mRNA levels (n≥5/group). *$p < 0.05$, **$p < 0.01$, ***$p < 0.001$. *Gnas*: Guanine nucleotide binding protein (G protein), alpha stimulating, *Prkaca*: Protein kinase CAMP-activated catalytic subunit alpha, *Gnai1*: G protein subunit alpha I1, *Adcy5*: Adenylate

cyclase 5, *Pik3r1*: Phosphoinositide-3-kinase regulatory subunit 1, *Itpka*: Inositol-trisphosphate 3-kinase A
